# Supplementary material for: Locally adaptive inversions in structured populations
Source: Genetics. 2024 May 6;227(3):iyae073. doi: 10.1093/genetics/iyae073 (PMC11979745; doi:10.1093/genetics/iyae073)

Locally adaptive inversions in structured populations

Carl Mackintosh, Michael F Scott, Max Reuter, Andrew Pomiankowski

This Mathematica notebook contains derivations for the results in the main text, and code used to generate the figures. The section titled “Preliminaries” should be run before the other sections (Mathematica should prompt you to do so in case you don’t!).

Preliminaries

This section contains useful substitutions, and functions that give approximations when the provided parameters are small and of the same order.

In[5]:=
```
subeqns2 = {p1g22 == 1 - p1g11 - p1g12 - p1g21, p1fA1 == p1g11 + p1g12,
   p1fB1 == p1g11 + p1g21, p1D == p1g11 p1g22 - p1g12 p1g21,
   p2g22 == 1 - p2g11 - p2g12 - p2g21, p2fA1 == p2g11 + p2g12,
   p2fB1 == p2g11 + p2g21, p2D == p2g11 p2g22 - p2g12 p2g21};
alleleSubs = Flatten[
   Solve[subeqns2, {p1g11, p1g12, p1g21, p1g22, p2g11, p2g12, p2g21, p2g22}]];
symmetrySubs = {m12 → m, m21 → m, s1 → s, s2 → s};
linearise[exp_, args_] :=
 With[{subs = Evaluate[Table[args⟦i⟧ → args⟦i⟧ ϵ, {i, 1, Length[args]}]]},
  Simplify[Normal[Series[exp /. subs, {ϵ, 0, 1}]] /. ϵ → 1]]
secondOrder[exp_, args_] :=
 With[{subs = Evaluate[Table[args⟦i⟧ → args⟦i⟧ ϵ, {i, 1, Length[args]}]]},
  Simplify[Normal[Series[exp /. subs, {ϵ, 0, 2}]] /. ϵ → 1]]
symmMiSubs = {m12 → m, m21 → m};
symmSelSubs = {s1 → s, s2 → s};
equilSubs = {p2g21 → p2g12, p1g21 → p1g12,
   p1g22 → 1 - 2 p1g12 - p1g11, p2g11 → 1 - 2 p2g12 - p2g22};
```

We use pigjk to refer to the frequency of genotype Aj Bk within deme i.

Continent-island model

Recursions

Step by step, we define our recursions. Prior to reproduction, adult frequencies are multiplied by

their relative fitness...

In[13]:=
```
ClearAll[p1w11, p1w12, p1w21, p1w22]
p1w11 = (1 + s) ^ 2;
p1w12 = (1 + s);
p1w21 = (1 + s);
p1w22 = 1 ;

Clear[p1Wm]
p1Wm = p1g11 p1w11 + p1g12 p1w12 + p1g21 p1w21 + p1g22 p1w22;

p1g11s = p1g11 p1w11 / p1Wm;
p1g12s = p1g12 p1w12 / p1Wm;
p1g21s = p1g21 p1w21 / p1Wm;
p1g22s = p1g22 p1w22 / p1Wm;
```

...so that their probability of reproduction can be incorporated into the reproduction phase.

In[24]:=
```
p1g11r =
  (1 - r) (p1g11s) + r (p1g11s ^ 2 + p1g11s p1g12s + p1g11s p1g21s + p1g12s p1g21s);
p1g12r =
  (1 - r) p1g12s + r (p1g12s ^ 2 + p1g12s p1g11s  + p1g12s p1g22s + p1g11s p1g22s);
p1g21r =
  (1 - r) p1g21s + r (p1g21s ^ 2 + p1g21s p1g11s + p1g21s p1g22s + p1g11s p1g22s);
p1g22r =
  (1 - r) p1g22s + r (p1g22s ^ 2 + p1g22s p1g12s + p1g22s p1g21s + p1g12s p1g21s);
```

All haplotype frequencies are reduced by migration, and then $A_2 B_2$ migrants are introduced.

In[28]:=
```
p1g11m = p1g11r (1 - m) ;
p1g12m = p1g12r (1 - m);
p1g21m = p1g21r (1 - m);
p1g22m = p1g22r (1 - m) + m;

p1g11m + p1g12m + p1g21m + p1g22m // Simplify
```

Out[32]=
```
1
```

The change in frequency is given by the difference between frequencies at the beginning of the generation, and the end of it (beginning of the next)

In[33]:=
```
CIdelp1g11 = Simplify[p1g11m - p1g11];
CIdelp1g12 = Simplify[p1g12m - p1g12];
CIdelp1g21 = Simplify[p1g21m - p1g21];
CIdelp1g22 = Simplify[p1g22m - p1g22];
```

Analytical approximations

In[37]:=
```
Dprime = p1g11m * p1g22m - p1g12m * p1g21m /. alleleSubs // Simplify;
delD = Dprime - p1D // Simplify;
```

Continuous time QLE

by assuming all rate parameters (m, s, r) are small and of the same order, we can derive a continuous time approximation to the discrete time system:

In[39]:=
```
dDdt = linearise[delD, {m, s, r}] // Simplify
```

Out[39]=

$$m\,p1fA1\,p1fB1 - p1D\,r - p1D\,(m + 2\,(-1 + p1fA1 + p1fB1)\,s)$$

find the equilibrium LD value in terms of allele frequencies:

In[40]:=
```
DsubAF = Solve[dDdt == 0, p1D]
```

Out[40]=

$$\left\{\left\{p1D \to \frac{m\,p1fA1\,p1fB1}{m + r - 2\,s + 2\,p1fA1\,s + 2\,p1fB1\,s}\right\}\right\}$$

now we repeat for the allele frequencies. since there is no difference between locus A and locus B, we can treat just one of them.

In[41]:=
```
dfA1dt =
 linearise[p1g11m + p1g12m - p1fA1 /. alleleSubs /. p1fB1 → p1fA1, {m, s, r}]
```

Out[41]=

$$-m\,p1fA1 + \left(p1D + p1fA1 - p1fA1^2\right)\,s$$

at QLE, m, s << r so that m/r, s/r << 1. therefore, we take 1/r as a small parameter in the approximation and solve to order 1/r, using the LD value we found earlier:

In[42]:=
```
Normal[Series[dfA1dt /. DsubAF /. p1fB1 → p1fA1, {r, Infinity, 1}]];
Solve[% == 0, p1fA1];
Collect[
 Simplify[Normal[Series[p1fA1 /. %[[2]], {r, Infinity, 1}]], r > 0 && s > 0], r]
```

Out[44]=

$$-\frac{m - s}{s} - \frac{m\,(m - s)}{r\,s}$$

now we can go back and find the equilibrium LD in terms of the model parameters

In[45]:=
```
Normal[Series[p1D /. DsubAF /. p1fB1 → p1fA1 /. p1fA1 → -(m - s)/s - m (m - s)/(r s),
   {r, Infinity, 1}]] // Simplify
```

Out[45]=

$$\left\{\frac{m\,(m - s)^2}{r\,s^2}\right\}$$

the initial rate of increase an inversion is then given by

In[46]:=
```
qleInvAdvCI = linearise[(p1g11m - p1g11) / p1g11 /. alleleSubs, {m, s, r}] /.
      p1g22 → 1 - p1g11 - p1g12 - p1g21 /. r → 0 // Simplify;
linearise[qleInvAdvCI /. alleleSubs /. p1fA1 → -(m - s)/s - m (m - s)/(r s) /.
   p1fB1 → -(m - s)/s - m (m - s)/(r s), {m, s, r}]
```

Out[47]=

$$\frac{m\,(2\,m + r - 2\,s)}{r}$$

In[48]:=
```
Reduce[D[m (m - s)^2/(r s^2), m] > 0 && s > 0 && m > 0 && r > 0]
```

Out[48]=

$$s > 0 \;\&\&\; \left(\left(0 < m < \frac{s}{3} \;\&\&\; r > 0\right) \;||\; (m > s \;\&\&\; r > 0)\right)$$

Continuous time via exact solution

Alternatively, we can find the advantage in the exact continuous time case and then check particular cases

In[49]:=
```
p1fA1cts = (p1fA1 /. Solve[(dfA1dt /. DsubAF /. p1fB1 → p1fA1) == 0, p1fA1]);

Quiet[CIadvcts =
  Flatten[linearise[(p1g11m - p1g11) / p1g11 /. r → 0 /. alleleSubs /. DsubAF /.
       p1fB1 → p1fA1 /. p1fA1 → p1fA1cts, {m, s, r}]][[3]]
]
```

Out[50]=

$$\frac{r\,s + 2\,s^2 - \sqrt{s^2\left(-8\,m\,r + (r + 2\,s)^2\right)}}{4\,s}$$

determine the advantage when i) migration is weak, ii) migration and selection are of the same order, iii) when recombination is strong (QLE)

In[51]:=
```
FullSimplify[linearise[CIadvcts, {m}], m > 0 && s > 0 && r > 0]
FullSimplify[linearise[CIadvcts, {m, s}], m > 0 && s > 0 && r > 0]
FullSimplify[Normal[Series[CIadvcts, {r, Infinity, 1}]], m > 0 && s > 0 && r > 0]
FullSimplify[
 Normal[Series[CIadvcts /. m → m ϵ /. s → s ϵ /. r → r ϵ, {ϵ, 0, 2}]] /. ϵ → 1,
 m > 0 && s > 0 && r > 0]
```

Out[51]=

$$\frac{m\,r}{r+2\,s}$$

Out[52]=

$$m$$

Out[53]=

$$\frac{m\,(2\,m+r-2\,s)}{r}$$

Out[54]=

$$\frac{1}{4}\left(r+2\,s-\sqrt{-8\,m\,r+(r+2\,s)^2}\right)$$

using the ‘reverse’ approximation 1 - x \approx 1/(1+x) when x is small, from the QLE expression we can recover equation A8 from Charlesworth & Barton 2018, Equation 13 in this manuscript (thanks to Denis Roze for pointing this out)

In[55]:=

$$\frac{m\,(2\,m+r-2\,s)}{r} - m\,(1-(2\,(s/r)-2\,(m/r)))\ \text{// Simplify}$$

Out[55]=

$$0$$

Numerical solution

In[56]:=
```
CIinvAdvNum[M_, S_, R_] := With[{freqs = FindRoot[
      {CIdelp1g11 == 0, CIdelp1g12 == 0, CIdelp1g21 == 0, CIdelp1g22 == 0} /. m → M /.
        r → R /. s → S, {{p1g11, 1}, {p1g12, 0.5}, {p1g21, 0.5}, {p1g22, 0}}]},
   (p1g11m - p1g11) / p1g11 /. r → 0 /. m → M /. s → S /. freqs]
```

Two deme model

Recursions

We proceed as in the continent-island model, but now include fitness and frequencies in deme 2:

In[57]:=
```
ClearAll[p1w11, p1w12, p1w21, p1w22, p2w11, p2w12, p2w21, p2w22]
wSubs = {p1w11 → (1 + s1) ^ 2 ,
   p1w12 → (1 + s1),
   p1w21 → (1 + s1),
   p1w22 → 1,
   p2w11 → 1,
   p2w12 → (1 + s2),
   p2w21 → (1 + s2),
   p2w22 → (1 + s2) ^ 2 };

ClearAll["p1Wm", "p2Wm"]
p1Wm = p1g11 p1w11 + p1g12 p1w12 + p1g21 p1w21 + p1g22 p1w22;
p2Wm = p2g11 p2w11 + p2g12 p2w12 + p2g21 p2w21 + p2g22 p2w22;

p1g11s = p1g11 p1w11 / p1Wm;
p1g12s = p1g12 p1w12 / p1Wm;
p1g21s = p1g21 p1w21 / p1Wm;
p1g22s = p1g22 p1w22 / p1Wm;

p2g11s = p2g11 p2w11 / p2Wm;
p2g12s = p2g12 p2w12 / p2Wm;
p2g21s = p2g21 p2w21 / p2Wm;
p2g22s = p2g22 p2w22 / p2Wm;
```

In[70]:=
```
p1g11r =
  (1 - r) (p1g11s) + r (p1g11s ^ 2 + p1g11s p1g12s + p1g11s p1g21s + p1g12s p1g21s);
p1g12r =
  (1 - r) p1g12s + r (p1g12s ^ 2 + p1g12s p1g11s + p1g12s p1g22s + p1g11s p1g22s);
p1g21r =
  (1 - r) p1g21s + r (p1g21s ^ 2 + p1g21s p1g11s + p1g21s p1g22s + p1g11s p1g22s);
p1g22r =
  (1 - r) p1g22s + r (p1g22s ^ 2 + p1g22s p1g12s + p1g22s p1g21s + p1g12s p1g21s);


p2g11r =
  (1 - r) (p2g11s) + r (p2g11s ^ 2 + p2g11s p2g12s + p2g11s p2g21s + p2g12s p2g21s);
p2g12r =
  (1 - r) p2g12s + r (p2g12s ^ 2 + p2g12s p2g11s + p2g12s p2g22s + p2g11s p2g22s);
p2g21r =
  (1 - r) p2g21s + r (p2g21s ^ 2 + p2g21s p2g11s + p2g21s p2g22s + p2g11s p2g22s);
p2g22r =
  (1 - r) p2g22s + r (p2g22s ^ 2 + p2g22s p2g12s + p2g22s p2g21s + p2g12s p2g21s);
```

In[78]:=
```
p1g11m = (p1g11r * (1 - m21) + m21 p2g11r);
p1g12m = (p1g12r * (1 - m21) + m21 p2g12r);
p1g21m = (p1g21r * (1 - m21) + m21 p2g21r);
p1g22m = (p1g22r * (1 - m21) + m21 p2g22r);
p2g11m = (p2g11r * (1 - m12) + m12 p1g11r);
p2g12m = (p2g12r * (1 - m12) + m12 p1g12r);
p2g21m = (p2g21r * (1 - m12) + m12 p1g21r);
p2g22m = (p2g22r * (1 - m12) + m12 p1g22r);

(p2g11m + p2g12m + p2g21m + p2g22m) // Simplify
(p1g11m + p1g12m + p1g21m + p1g22m) // Simplify
```

Out[86]=
```
1
```

Out[87]=
```
1
```

In[88]:=
```
delp1g11 = Simplify[p1g11m - p1g11];
delp1g12 = Simplify[p1g12m - p1g12];
delp1g21 = Simplify[p1g21m - p1g21];
delp1g22 = Simplify[p1g22m - p1g22];

delp2g11 = Simplify[p2g11m - p2g11];
delp2g12 = Simplify[p2g12m - p2g12];
delp2g21 = Simplify[p2g21m - p2g21];
delp2g22 = Simplify[p2g22m - p2g22];
```

In[96]:=
```
postSelectionSubs =
  {D1s → (s1g11 s1g22 - s1g12 s1g21), D2s → (s2g11 s2g22 - s2g12 s2g21),
   s1g11 → p1g11 p1w11 / wbar1, s1g12 → p1g12 p1w12 / wbar1,
   s1g21 → p1g21 p1w21 / wbar1, s1g22 → p1g22 p1w22 / wbar1,
   s2g11 → p2g11 p2w11 / wbar2, s2g12 → p2g12 p2w12 / wbar2,
   s2g21 → p2g21 p2w21 / wbar2, s2g22 → p2g22 p2w22 / wbar2};
```

Verify that the final recursions can be written in the simplified form given in the main text:

In[97]:=
```
p1X11 = ((1 - m21) (s1g11 - r D1s) + m21 (s2g11 - r D2s));
p1X12 = ((1 - m21) (s1g12 + r D1s) + m21 (s2g12 + r D2s));
p1X21 = ((1 - m21) (s1g21 + r D1s) + m21 (s2g21 + r D2s));
p1X22 = ((1 - m21) (s1g22 - r D1s) + m21 (s2g22 - r D2s));
p2X11 = ((1 - m12) (s2g11 - r D2s) + m12 (s1g11 - r D1s));
p2X12 = ((1 - m12) (s2g12 + r D2s) + m12 (s1g12 + r D1s));
p2X21 = ((1 - m12) (s2g21 + r D2s) + m12 (s1g21 + r D1s));
p2X22 = ((1 - m12) (s2g22 - r D2s) + m12 (s1g22 - r D1s));
recursions = {p1g11m, p1g12m, p1g21m, p1g22m, p2g11m, p2g12m, p2g21m, p2g22m};
simplifiedRecursions = {p1X11, p1X12, p1X21, p1X22, p2X11, p2X12, p2X21, p2X22};
(recursions - simplifiedRecursions ) /. postSelectionSubs /. postSelectionSubs /.
   wbar1 → p1Wm /. wbar2 → p2Wm // Simplify
```

Out[107]=
```
{0, 0, 0, 0, 0, 0, 0, 0}
```

The following matrix corresponds to C_{11} as written in the main text :

In[108]:=

```
mat = {{(1 - m21) ((1 + s1)^2)/wbar1, m12 ((1 + s1)^2)/wbar1}, {m21/wbar2, (1 - m12)/wbar2}};
charpoly = Simplify[Det[mat - IdentityMatrix[2] * λ]];
```

Numerical

Or, we can solve for the allele freq

In[110]:=

```
twoDemeHapFreqsNum[M12_, M21_, S1_, S2_, R_] :=
 FindRoot[{delp1g11 == 0, delp1g12 == 0, delp1g21 == 0, delp1g22 == 0,
          delp2g11 == 0, delp2g12 == 0, delp2g21 == 0, delp2g22 == 0} /. wSubs /.
        m12 → M12 /. m21 → M21 /. s1 → S1 /. s2 → S2 /. r → R,
   {{p1g11, 1}, {p1g12, 0.5}, {p1g21, 0.5}, {p1g22, 0}, {p2g11, 0},
    {p2g12, 0.5}, {p2g21, 0.5}, {p2g22, 1}}]

twoDemeInvAdvNum[M12_, M21_, S1_, S2_, R_] :=
 With[{freqs = FindRoot[{delp1g11 == 0, delp1g12 == 0, delp1g21 == 0, delp1g22 == 0,
             delp2g11 == 0, delp2g12 == 0, delp2g21 == 0, delp2g22 == 0} /. wSubs /.
           m12 → M12 /. m21 → M21 /. s1 → S1 /. s2 → S2 /. r → R,
      {{p1g11, 1}, {p1g12, 0.5}, {p1g21, 0.5}, {p1g22, 0}, {p2g11, 0},
       {p2g12, 0.5}, {p2g21, 0.5}, {p2g22, 1}}]},

  Max[
    λ /. Solve[(charpoly /. wbar1 → p1Wm /. wbar2 → p2Wm /. wSubs /. m12 → M12 /. m21 →
              M21 /. s1 → S1 /. s2 → S2 /. r → R /. freqs) == 0, λ]] - 1]
```

Analytical approximations

Equilibrium allele frequencies

in the QLE approximation, we assume that r >> m, s, so that m/r, s/r << 1. so, we take 1/r as a small parameter in the model. this method follows that of Akerman and Buerger 2014.

so, the allele frequencies at QLE are of the form f1A1 = F0 + F1/r + O(1/r^2) etc.

first, we convert the model to continuous time

In[112]:=

```
df1A1 =
  linearise[p1g11m + p1g12m - p1fA1 /. alleleSubs /. wSubs, {m12, m21, s1, s2, r}];
df2A1 =
  linearise[p2g11m + p2g12m - p2fA1 /. alleleSubs /. wSubs, {m12, m21, s1, s2, r}];
df1B1 =
  linearise[p1g11m + p1g21m - p1fB1 /. alleleSubs /. wSubs, {m12, m21, s1, s2, r}];
df2B1 =
  linearise[p2g11m + p2g21m - p2fB1 /. alleleSubs /. wSubs, {m12, m21, s1, s2, r}];
dD1 = linearise[p1g11m p1g22m - p1g12m p1g21m - p1D /. alleleSubs /. wSubs,
   {m12, m21, s1, s2, r}];
dD2 = linearise[p2g11m p2g22m - p2g12m p2g21m - p2D /. alleleSubs /. wSubs,
   {m12, m21, s1, s2, r}];
rsubs = {p1fA1 → f10 + f11 / r,
  p2fA1 → f20 + f21 / r, p1fB1 → g10 + g11 / r , p2fB1 → g20 + g21 / r}
```

Out[118]=

$$\left\{p1fA1 \to f10 + \frac{f11}{r},\ p2fA1 \to f20 + \frac{f21}{r},\ p1fB1 \to g10 + \frac{g11}{r},\ p2fB1 \to g20 + \frac{g21}{r}\right\}$$

at QLE, close to D = 0, the rate of generation of LD due to selection is equal to the rate of decay caused by recombination:

In[119]:=

```
p1D /. Solve[(dD1 /. p1D → 0 /. p2D → 0) == r p1D, p1D] // Simplify;
p2D /. Solve[(dD2 /. p1D → 0 /. p2D → 0) == r p2D, p2D] // Simplify;
{%%, %} /. rsubs;
Coefficient[%, 1 / r] / r // Simplify;
{D1sub, D2sub} = % // Flatten
```

Out[123]=

$$\left\{\frac{(f10 - f20)\ (g10 - g20)\ m21}{r},\ \frac{(f10 - f20)\ (g10 - g20)\ m12}{r}\right\}$$

at equilibrium the following expressions are equal to 0

In[124]:=

```
dAlleleFreq = {df1A1, df2A1 , df1B1, df2B1};
```

using this value of LD, determine the linear term in the allele frequencies, which are the allele frequencies when the system is in full linkage equilibrium (ie D = 0)

In[125]:=

```
dAlleleFreq /. rsubs /. p1D → D1sub /. p2D → D2sub // Simplify;
% /. r → 1 / R // Simplify;
% /. R → 0;
freqLinearTermSubs = Simplify[Solve[% == 0, {f10, f20, g10, g20}][[16]]]
```

Out[128]=

$$\left\{f10 \to \frac{1}{2}\left(1 - \frac{2\ m21}{s1} + \frac{\sqrt{4\ m12\ m21 + s1\ s2}}{\sqrt{s1}\ \sqrt{s2}}\right),\ f20 \to \frac{2\ m12 + s2 - \frac{\sqrt{s2}\ \sqrt{4\ m12\ m21 + s1\ s2}}{\sqrt{s1}}}{2\ s2},\right.$$

$$\left. g10 \to \frac{1}{2}\left(1 - \frac{2\ m21}{s1} + \frac{\sqrt{4\ m12\ m21 + s1\ s2}}{\sqrt{s1}\ \sqrt{s2}}\right),\ g20 \to \frac{2\ m12 + s2 - \frac{\sqrt{s2}\ \sqrt{4\ m12\ m21 + s1\ s2}}{\sqrt{s1}}}{2\ s2}\right\}$$

now find the O(1/r) term

In[129]:=

```
dAlleleFreq /. rsubs /. p1D → D1sub /. p2D → D2sub // Simplify;
% /. r → 1 / R // Simplify;
D[%, R] /. R → 0 // Simplify
freqFirstOrderTermSubs = Simplify[Solve[% == 0, {f11, f21, g11, g21}]] // Flatten
```

Out[131]=

$$\{f11\,(-m21+s1-2\,f10\,s1)+m21\,(f21+(f10-f20)\,(g10-g20)\,s1),\; f11\,m12-(f10-f20)\,(g10-g20)\,m12\,s2-f21\,(m12+s2-2\,f20\,s2),\; g11\,(-m21+s1-2\,g10\,s1)+m21\,(g21+(f10-f20)\,(g10-g20)\,s1),\; g11\,m12-(f10-f20)\,(g10-g20)\,m12\,s2-g21\,(m12+s2-2\,g20\,s2)\}$$

Out[132]=

$$\Big\{f11\to\frac{(f10-f20)\,(g10-g20)\,m21\,((-1+2\,f20)\,s1\,s2+m12\,(-s1+s2))}{m12\,(s1-2\,f10\,s1)+(-1+2\,f20)\,(m21+(-1+2\,f10)\,s1)\,s2},$$
$$f21\to\frac{(f10-f20)\,(g10-g20)\,m12\,((-1+2\,f10)\,s1\,s2+m21\,(-s1+s2))}{m12\,(s1-2\,f10\,s1)+(-1+2\,f20)\,(m21+(-1+2\,f10)\,s1)\,s2},$$
$$g11\to\frac{(f10-f20)\,(g10-g20)\,m21\,((-1+2\,g20)\,s1\,s2+m12\,(-s1+s2))}{m12\,(s1-2\,g10\,s1)+(-1+2\,g20)\,(m21+(-1+2\,g10)\,s1)\,s2},$$
$$g21\to\frac{(f10-f20)\,(g10-g20)\,m12\,((-1+2\,g10)\,s1\,s2+m21\,(-s1+s2))}{m12\,(s1-2\,g10\,s1)+(-1+2\,g20)\,(m21+(-1+2\,g10)\,s1)\,s2}\Big\}$$

then we can find the value of LD at the QLE:

In[133]:=

```
qleAlleleFreqs = Simplify[rsubs /. freqFirstOrderTermSubs /. freqLinearTermSubs,
    s1 > 0 && s2 > 0 && m12 > 0 && m21 > 0 && r > 0] // Flatten;
qleD = FullSimplify[{p1D → D1sub, p2D → D2sub} /. freqLinearTermSubs,
   s1 > 0 && s2 > 0 && m12 > 0 && m21 > 0]
```

Out[134]=

$$\Big\{p1D\to\frac{m21\,\left(m12\,s1+m21\,s2-\sqrt{s1\,s2\,(4\,m12\,m21+s1\,s2)}\right)^2}{r\,s1^2\,s2^2},$$
$$p2D\to\frac{m12\,\left(m12\,s1+m21\,s2-\sqrt{s1\,s2\,(4\,m12\,m21+s1\,s2)}\right)^2}{r\,s1^2\,s2^2}\Big\}$$

Find the conditions under which it increases:

In[135]:=
```
FullSimplify[D1sub /. freqLinearTermSubs, s1 > 0 && s2 > 0 && m12 > 0 && m21 > 0]
Simplify[% /. symmetrySubs, s > 0 && r > 0 && m > 0]
Reduce[D[%, m] > 0 && s > 0 && m > 0 && r > 0]
```

Out[135]=

$$\frac{m21\left(m12\ s1 + m21\ s2 - \sqrt{s1\ s2\ (4\ m12\ m21 + s1\ s2)}\right)^2}{r\ s1^2\ s2^2}$$

Out[136]=

$$\frac{m\left(-2\ m + \sqrt{4\ m^2 + s^2}\right)^2}{r\ s^2}$$

Out[137]=

$$s > 0\ \&\&\ 0 < m < \frac{\sqrt{s^2}}{2\sqrt{3}}\ \&\&\ r > 0$$

In[138]:=
```
simpleLinearTerms = Simplify[
  freqLinearTermSubs /. m12 → s2 l2 /. m21 → s1 l1, s1 > 0 && s2 > 0 && l1 > 0 && l2 > 0]
Simplify[freqFirstOrderTermSubs /. m12 → s2 l2 /. m21 → s1 l1,
  s1 > 0 && s2 > 0 && l1 > 0 && l2 > 0];
{f11 / (g10 - g20) /. % /. simpleLinearTerms,
  f21 / (g10 - g20) /. % /. simpleLinearTerms,
  g11 / (f10 - f20) /. % /. simpleLinearTerms,
  g21 / (f10 - f20) /. % /. simpleLinearTerms} // Simplify
```

Out[138]=

$$\left\{f10 \to \frac{1}{2}\left(1 - 2\ l1 + \sqrt{1 + 4\ l1\ l2}\right),\ f20 \to \frac{1}{2} + l2 - \frac{1}{2}\sqrt{1 + 4\ l1\ l2},\right.$$
$$\left. g10 \to \frac{1}{2}\left(1 - 2\ l1 + \sqrt{1 + 4\ l1\ l2}\right),\ g20 \to \frac{1}{2} + l2 - \frac{1}{2}\sqrt{1 + 4\ l1\ l2}\right\}$$

Out[140]=

$$\left\{l1\left(s1 - \frac{l2\ (s1 + s2)}{\sqrt{1 + 4\ l1\ l2}}\right),\ -l2\ s2 + \frac{l1\ l2\ (s1 + s2)}{\sqrt{1 + 4\ l1\ l2}},\right.$$
$$\left. l1\left(s1 - \frac{l2\ (s1 + s2)}{\sqrt{1 + 4\ l1\ l2}}\right),\ -l2\ s2 + \frac{l1\ l2\ (s1 + s2)}{\sqrt{1 + 4\ l1\ l2}}\right\}$$

Initial rate of increase

find an approximation for the eigenvalue at QLE:

In[141]:=
```
qleEvals =
  Solve[(charpoly /. wbar1 → p1Wm /. wbar2 → p2Wm /. wSubs /. alleleSubs /. rsubs /.
       freqFirstOrderTermSubs /.
     freqLinearTermSubs /. qleD /. symmetrySubs) == 0, λ];
```

In[142]:=

```
approxEvals = FullSimplify[
  Normal[Series[λ /. qleEvals /. symmetrySubs /. p2D → p1D /. r → r ϵ /. m → m ϵ^2 /.
     s → s ϵ^2, {ϵ, 0, 3}]] /. ϵ → 1, {s > 0, m > 0}]
```

Out[142]=

$$\left\{1+m+\frac{8\,m^2}{r}-\sqrt{m^2+s^2}-\sqrt{4\,m^2+s^2}-\frac{2\,m\,(8\,m^2+s^2)}{r\,\sqrt{4\,m^2+s^2}},\ 1+m+\frac{8\,m^2}{r}+\sqrt{m^2+s^2}-\sqrt{4\,m^2+s^2}-\frac{2\,m\,(8\,m^2+s^2)}{r\,\sqrt{4\,m^2+s^2}}\right\}$$

In[143]:=

```
FullSimplify[
 Normal[Series[λ /. qleEvals /. symmetrySubs /. p2D → p1D /. r → r ϵ /. m → m ϵ /.
    s → s ϵ, {ϵ, 0, 1}]] /. ϵ → 1, {s > 0, m > 0, r > 0}]
FullSimplify[Normal[Series[%, {r, Infinity, 1}]], {l > 0, m > 0, r > 0}]
```

Out[143]=

$$\left\{1+m+\frac{8\,m^2}{r}-\sqrt{m^2+s^2}-\sqrt{4\,m^2+s^2}-\frac{2\,m\,(8\,m^2+s^2)}{r\,\sqrt{4\,m^2+s^2}},\ 1+m+\frac{8\,m^2}{r}+\sqrt{m^2+s^2}-\sqrt{4\,m^2+s^2}-\frac{2\,m\,(8\,m^2+s^2)}{r\,\sqrt{4\,m^2+s^2}}\right\}$$

Out[144]=

$$\left\{1+m-\sqrt{m^2+s^2}-\sqrt{4\,m^2+s^2}+\frac{8\,m^2-\frac{2\,m\,(8\,m^2+s^2)}{\sqrt{4\,m^2+s^2}}}{r},\ 1+m+\sqrt{m^2+s^2}-\sqrt{4\,m^2+s^2}+\frac{8\,m^2-\frac{2\,m\,(8\,m^2+s^2)}{\sqrt{4\,m^2+s^2}}}{r}\right\}$$

Show the result can be written as in eqn 14 of the main text:

In[145]:=

```
Simplify[(1 + m - s (A - Sqrt[1 + α^2]) - (m / r) (s (4 A^2 - 2) / A - 8 m) /.
    A → Sqrt[1 + 4 α^2]) - (approxEvals⟦2⟧) /. α → m / s, m > 0 && s > 0]
```

Out[145]=

0

Show further how this result can be written in terms of genetic load:

In[146]:=

```
linearise[p1Wm /. wSubs /. alleleSubs /. qleD /. rsubs /. freqFirstOrderTermSubs /.
   freqLinearTermSubs /. symmetrySubs, {m, s, r}];
linearise[1 - % / (1 + s) ^2, {m, s, r}];
p1Load = Normal[Series[%, {r, Infinity, 1}]] // Simplify;
1 + p1Load - (m + s) + √(m^2 + s^2) - (approxEvals⟦2⟧) // FullSimplify
```

Out[149]=

0

In the limit of weak migration, this can be written L - m, similar to Proulx & Teotonio 2022, Proulx & Philips 2005

In[150]:=

```
linearise[p1Wm /. wSubs /. alleleSubs /. qleD /. rsubs /. freqFirstOrderTermSubs /.
    freqLinearTermSubs /. symmetrySubs, {m, s, r}];
linearise[1 - % / (1 + s) ^ 2, {m, s, r}];
p1LoadWeakm = Simplify[Normal[Series[%, {m, 0, 1}]], s > 0] // Simplify;
Simplify[Normal[Series[approxEvals[[2]], {m, 0, 1}]], s > 0];
1 + p1LoadWeakm - m - (%) // FullSimplify
```

Out[154]=

```
0
```

Numerically solved invasion probability

Numerically solve the simultaneous equations from the branching process and calculate the invasion probability given capture, and the weighted probability once we also include the probability of capture (establishment).

In[155]:=

```
branchinginv1[M12_, M21_, S1_, S2_, R_] :=

 If[Max[M12, M21] < Min[S1, S2],
  With[{freqs = Flatten[twoDemeHapFreqsNum[M12, M21, S1, S2, R]]},
   ((1 - z1)) (p1g11) / (p1g11 + p2g11) + ((1 - z2)) (p2g11) / (p1g11 + p2g11) /.
      equilSubs /. freqs /. FindRoot[
     ({Exp[-(mat⟦1, 1⟧ (1 - z1) + mat⟦1, 2⟧ (1 - z2))], Exp[-(mat⟦2, 1⟧ (1 - z1) +
                        mat⟦2, 2⟧ (1 - z2))]} /. wbar1 → p1Wm /. wbar2 → p2Wm /.
                wSubs /. m12 → M12 /. m21 → M21 /. s1 → S1 /. s2 → S2 /. r → R /.
          equilSubs /. equilSubs /. freqs) == {z1, z2}, {{z1, 0}, {z2, 0}}]], Null]

branchinginv1weighted[M12_, M21_, S1_, S2_, R_] :=

 If[Max[M12, M21] < Min[S1, S2],
  With[{freqs = Flatten[twoDemeHapFreqsNum[M12, M21, S1, S2, R]]},
   ((1 - z1) * p1g11 + (1 - z2) * p2g11) /. FindRoot[
       ({Exp[-(mat⟦1, 1⟧ (1 - z1) + mat⟦1, 2⟧ (1 - z2))], Exp[-(mat⟦2, 1⟧ (1 - z1) +
                        mat⟦2, 2⟧ (1 - z2))]} /. wbar1 → p1Wm /. wbar2 → p2Wm /.
                wSubs /. m12 → M12 /. m21 → M21 /. s1 → S1 /. s2 → S2 /.
           r → R /. equilSubs /. equilSubs /. freqs) == {z1, z2},
       {{z1, 0}, {z2, 0}}] /. equilSubs /. freqs], Null]

branchinginv2weighted[M12_, M21_, S1_, S2_, R_] :=

 If[Max[M12, M21] < Min[S1, S2],
  With[{freqs = Flatten[twoDemeHapFreqsNum[M12, M21, S1, S2, R]]},
   ((1 - z1) * p1g22 + (1 - z2) * p2g22) /.
      FindRoot[({Exp[-(mat2⟦1, 1⟧ (1 - z1) + mat2⟦1, 2⟧ (1 - z2))],
                   Exp[-(mat2⟦2, 1⟧ (1 - z1) + mat2⟦2, 2⟧ (1 - z2))]} /. wbar1 →
                   p1Wm /. wbar2 → p2Wm /. wSubs /. m12 → M12 /. m21 → M21 /. s1 →
                S1 /. s2 → S2 /. r → R /. equilSubs /. equilSubs /. freqs) ==
        {z1, z2}, {{z1, 0}, {z2, 0}}] /. equilSubs /. freqs], Null]
```

Figures

Fig 1

In[158]:=

```
twoDemeSymmPlot1 =
  DiscretePlot[{branchinginv1[0.001, 0.001, S, S, 0.01], branchinginv1[
     0.001, 0.001, S, S, 0.05], branchinginv1[0.001, 0.001, S, S, 0.15]},
   {S, 0.001, 0.05, 0.0005}, PlotRange → {0, 0.0025}, Filling → None,
   Joined → True, PlotStyle → {{Black, Dashed, Thick}, {Blue, Dashed, Thick},
     {Red, Dashed, Thick}}, AxesLabel → {Style[Text["s"], FontSize → 12],
     Style[Text["invasion \nprobability"], FontSize → 12]},
   TicksStyle → Directive["Label", 10], AxesStyle → Thick,
   ImageSize → Small, Ticks → {{0.001, 0.001}, 0.002, 0.003},
   Epilog → {Text[Style["A", 14, Black], {0.0035, 0.0023}]}];


CIplt1 = DiscretePlot[{branchinginv1[0, 0.001, S, S, 0.01],
    branchinginv1[0, 0.001, S, S, 0.05], branchinginv1[0, 0.001, S, S, 0.15]},
   {S, 0.001, 0.05, 0.0005}, PlotRange → {0, 0.0025}, Filling → None,
   Joined → True, AxesLabel → {"s", "invasion probability"},
   PlotStyle → {{Black, Thick}, {Blue, Thick}, {Red, Thick}},
   AxesStyle → Thick, TicksStyle → Directive["Label", 10]];

twoDemeSymmPlot2 = DiscretePlot[{branchinginv1[0.01, 0.01, S, S, 0.01],
    branchinginv1[0.01, 0.01, S, S, 0.05], branchinginv1[0.01, 0.01, S, S, 0.15]},
   {S, 0.01, 0.05, 0.0005}, PlotRange → {0, 0.025}, Filling → None,
   Joined → True, PlotStyle → {{Black, Dashed, Thick},
     {Blue, Dashed, Thick}, {Red, Dashed, Thick}}, AxesLabel →
    {Style[Text["s"], FontSize → 12], Style[Text[" \n"], FontSize → 12]},
   TicksStyle → Directive["Label", 10], AxesStyle → Thick, ImageSize → Small,
   Epilog → {Text[Style["B", 14, Black], {0.013, 0.023}]}];


CIplt2 = DiscretePlot[{branchinginv1[0, 0.01, S, S, 0.01],
    branchinginv1[0, 0.01, S, S, 0.05], branchinginv1[0, 0.01, S, S, 0.15]},
   {S, 0.01, 0.05, 0.0005}, PlotRange → {0, 0.025}, Filling → None,
   Joined → True, AxesLabel → {"s", ""},
   PlotStyle → {{Black, Thick}, {Blue, Thick}, {Red, Thick}},
   AxesStyle → Thick, TicksStyle → Directive["Label", 10]];

Grid[{{Show[{twoDemeSymmPlot1, CIplt1}],
   Show[{twoDemeSymmPlot2, CIplt2}](*,Show[{twoDemeSymmPlot3,CIplt3}]*)}, {}}]
```

Out[162]=

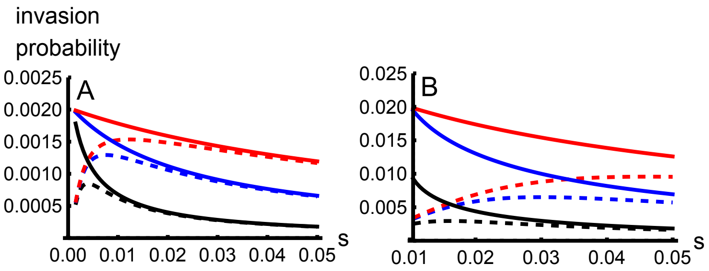
invasion
probability
0.0025
0.0020
0.0015
0.0010
0.0005
A
0.00 0.01 0.02 0.03 0.04 0.05
s
0.025
0.020
0.015
0.010
0.005
B
0.01 0.02 0.03 0.04 0.05
s

Fig 2

In[163]:=

```
Quiet[
 fig2A = ListContourPlot[
   Flatten[Table[{S1, M21, branchinginv1[0.01, M21, S1, 0.03, 0.5]},
     {S1, 0.008, 0.05, 0.002}, {M21, 0.0, 0.0325, 0.00025}], 1],
   Contours → Table[i / 400, {i, 0, 20}], ColorFunction →
    ColorData[{"BlueGreenYellow", {0, 0.05}}],
   ColorFunctionScaling → False, PlotLegends → None, ImageSize → Small,
   Epilog → {Text[Style["A", 14, Black], {0.0135, 0.02825}]},
   FrameLabel → {Style[Text["s1"], FontSize → 12],
     Style[Text["m21"], FontSize → 12], None, None}, FrameTicks →
    {{{0, 0.01, 0.02, 0.03}, None}, {{0, 0.01, 0.02, 0.03, 0.04, 0.05}, None}}];
 fig2B = ListContourPlot[
   Flatten[Table[{M12, M21, branchinginv1[M12, M21, 0.03, 0.03, 0.5]},
     {M12, 0.0, 0.0325, 0.00025}, {M21, 0.0, 0.0325, 0.00025}], 1],
   Contours → Table[i / 400, {i, 0, 20}], ColorFunction →
    ColorData[{"BlueGreenYellow", {0, 0.05}}],
   ColorFunctionScaling → False, PlotLegends → None, ImageSize → Small,
   Epilog → {Text[Style["B", 14, Black], {0.0014, 0.028}]},
   FrameLabel → {Style[Text["m12"], FontSize → 12],
     Style[Text["m21"], FontSize → 12], None, None},
   FrameTicks → {{{0, 0.01, 0.02, 0.03}, None}, {{0, 0.01, 0.02, 0.03}, None}}];
 fig2C =
  ListContourPlot[Flatten[Table[{S1, S2, branchinginv1[0.01, 0.01, S1, S2, 0.5]},
     {S1, 0.01, 0.05, 0.001}, {S2, 0.01, 0.05, 0.001}], 1],
   Contours → Table[i / 400, {i, 0, 20}], ColorFunction →
    ColorData[{"BlueGreenYellow", {0, 0.05}}],
   ColorFunctionScaling → False, PlotLegends → None, ImageSize → Small,
   Epilog → {Text[Style["C", 14, White], {0.013, 0.0475}]},
   FrameLabel → {Style[Text["s1"], FontSize → 12],
     Style[Text["s2"], FontSize → 12], None, None},
   FrameTicks → {{{0, 0.01, 0.02, 0.03, 0.04, 0.05}, None},
     {{0, 0.01, 0.02, 0.03, 0.04, 0.05}, None}}];
 fig2D = ListContourPlot[
   Flatten[Table[{S2, M21, branchinginv1[0.01, M21, 0.03, S2, 0.5]},
     {S2, 0.01, 0.05, 0.001}, {M21, 0.0, 0.0325, 0.0001}], 1],
   Contours → Table[i / 400, {i, 0, 20}], ColorFunction →
    ColorData[{"BlueGreenYellow", {0, 0.05}}],
   ColorFunctionScaling → False, PlotLegends → None, ImageSize → Small,
   Epilog → {Text[Style["D", 14, Black], {0.013, 0.02825}]},
   FrameLabel → {Style[Text["s2"], FontSize → 12],
     Style[Text["m21"], FontSize → 12], None, None}, FrameTicks →
    {{{0, 0.01, 0.02, 0.03}, None}, {{0, 0.01, 0.02, 0.03, 0.04, 0.05}, None}}];]
```

Note: the Ticks option is supposedly deprecated, but still works here...

In[164]:=

```
legend =
  BarLegend[{"BlueGreenYellow", {-0.0001, 0.05001}}, Table[i / 400, {i, 0, 20}],
   LegendMarkerSize → 300, LegendLabel → "invasion probability",
   Ticks → {0, 0.01, 0.02, 0.03, 0.04, 0.05}];
Legended[Grid[{{fig2A,
     fig2B}, {fig2C,
     fig2D}}], legend]
```

Out[165]=

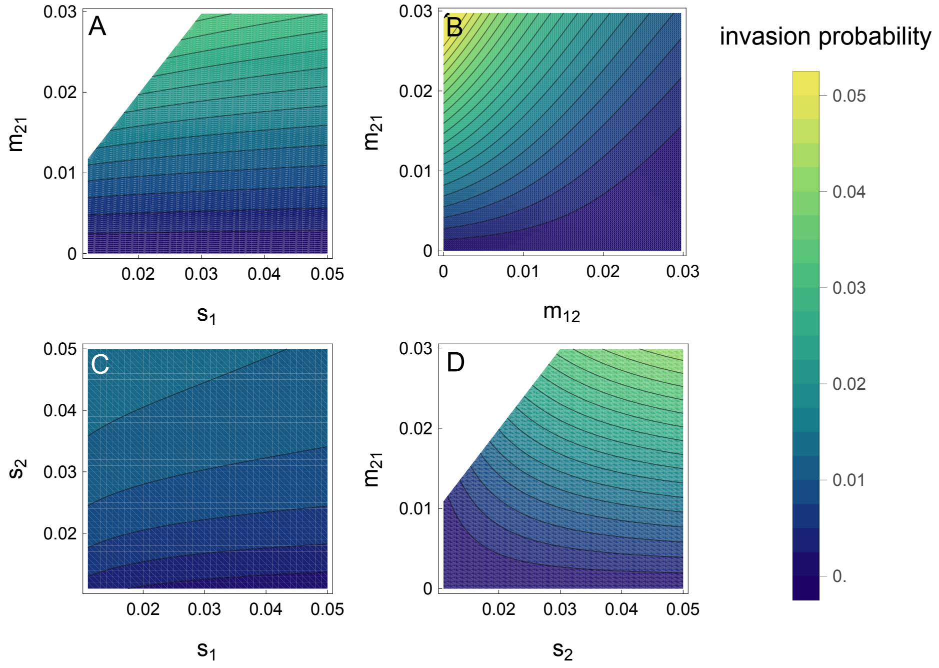

Fig 3

In[166]:=

```
twoDemeSymmPlot3 = DiscretePlot[{branchinginv1weighted[0.001, 0.001, S, S, 0.01],
     branchinginv1weighted[0.001, 0.001, S, S, 0.05],
     branchinginv1weighted[0.001, 0.001, S, S, 0.15]},
    {S, 0.001, 0.05, 0.0005}, PlotRange → {0, 0.0018}, Filling → None,
    Joined → True, PlotStyle → {{Black, Dashed, Thick},
      {Blue, Dashed, Thick}, {Red, Dashed, Thick}}, AxesLabel →
     {Style[Text["s"], FontSize → 12], Style[Text["γ11"], FontSize → 12]},
    TicksStyle → Directive["Label", 10], AxesStyle → Thick,
    ImageSize → Small, Ticks → {{0.001, 0.001}, 0.002, 0.003},
    Epilog → {Text[Style["A", 14, Black], {0.004, 0.0016}]}];


CIplt3 = DiscretePlot[
    {branchinginv1weighted[0, 0.001, S, S, 0.01], branchinginv1weighted[
      0, 0.001, S, S, 0.05], branchinginv1weighted[0, 0.001, S, S, 0.15]},
    {S, 0.001, 0.05, 0.0005}, PlotRange → {0, 0.0018}, Filling → None,
    Joined → True, AxesLabel → {"s", "invasion probability"},
    PlotStyle → {{Black, Thick}, {Blue, Thick}, {Red, Thick}},
    AxesStyle → Thick, TicksStyle → Directive["Label", 10],
    Epilog → {Text[Style["A", 18, Black], {0.01, 0.0015}]}];


twoDemeSymmPlot4 = DiscretePlot[{branchinginv1weighted[0.01, 0.01, S, S, 0.01],
     branchinginv1weighted[0.01, 0.01, S, S, 0.05],
     branchinginv1weighted[0.01, 0.01, S, S, 0.15]}, {S, 0.01, 0.05, 0.0005},
    PlotRange → {0, 0.018}, Filling → None, Joined → True, PlotStyle →
     {{Black, Dashed, Thick}, {Blue, Dashed, Thick}, {Red, Dashed, Thick}},
    AxesLabel → {Style[Text["s"], FontSize → 12], Style[Text[" "], FontSize → 12]},
    TicksStyle → Directive["Label", 10], AxesStyle → Thick, ImageSize → Small,
    Epilog → {Text[Style["B", 14, Black], {0.0135, 0.016}]}];


CIplt4 = DiscretePlot[{branchinginv1weighted[0, 0.01, S, S, 0.01],
     branchinginv1weighted[0, 0.01, S, S, 0.05],
     branchinginv1weighted[0, 0.01, S, S, 0.15]}, {S, 0.01, 0.05, 0.0005},
    PlotRange → {0, 0.018}, Filling → None, Joined → True, AxesLabel → {"s", ""},
    PlotStyle → {{Black, Thick}, {Blue, Thick}, {Red, Thick}},
    AxesStyle → Thick, TicksStyle → Directive["Label", 10]];
Grid[{{Show[{twoDemeSymmPlot3, CIplt3}], Show[{twoDemeSymmPlot4, CIplt4}]}, {}}]
```

Out[170]=

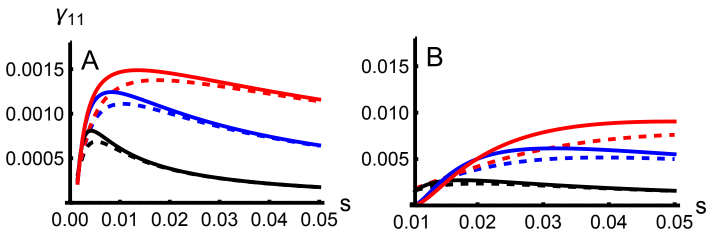

Fig 4

In[172]:=

```
Quiet[
 fig4A = ListContourPlot[
   Flatten[Table[{M12, M21, branchinginv1weighted[M12, M21, 0.03, 0.03, 0.1]},
     {M12, 0.0, 0.0325, 0.00025}, {M21, 0.0, 0.0325, 0.00025}], 1],
   Contours → Table[i / 800, {i, 0, 12}], ColorFunction →
    ColorData[{"BlueGreenYellow", {0, 0.015}}],
   ColorFunctionScaling → False, PlotLegends → None, ImageSize → Small,
   Epilog → {Text[Style["A", 14, White], {0.0014, 0.028}]},
   FrameLabel → {Style[Text["m12"], FontSize → 12],
     Style[Text["m21"], FontSize → 12], None, None},
   FrameTicks → {{{0, 0.01, 0.02, 0.03}, None}, {{0, 0.01, 0.02, 0.03}, None}}];

 fig4B = ListContourPlot[
   Flatten[Table[{S1, S2, branchinginv1weighted[0.01, 0.01, S1, S2, 0.1]},
     {S1, 0.01, 0.05, 0.001}, {S2, 0.01, 0.05, 0.001}], 1],
   Contours → Table[i / 800, {i, 0, 12}], ColorFunction →
    ColorData[{"BlueGreenYellow", {0, 0.015}}],
   ColorFunctionScaling → False, PlotLegends → None, ImageSize → Small,
   Epilog → {Text[Style["B", 14, White], {0.013, 0.0475}]},
   FrameLabel → {Style[Text["s1"], FontSize → 12],
     Style[Text["s2"], FontSize → 12], None, None},
   FrameTicks → {{{0, 0.01, 0.02, 0.03, 0.04, 0.05}, None},
     {{0, 0.01, 0.02, 0.03, 0.04, 0.05}, None}}];

 fig4C = ListContourPlot[
   Flatten[Table[{M12, M21, branchinginv1weighted[M12, M21, 0.03, 0.03, 0.1] +
       branchinginv2weighted[M12, M21, 0.03, 0.03, 0.1]},
     {M12, 0.0, 0.0325, 0.00025}, {M21, 0.0, 0.0325, 0.00025}], 1],
   Contours → Table[i / 800, {i, 0, 12}], ColorFunction →
    ColorData[{"BlueGreenYellow", {0, 0.015}}],
   ColorFunctionScaling → False, PlotLegends → None, ImageSize → Small,
   Epilog → {Text[Style["C", 14, White], {0.0014, 0.028}]},
   FrameLabel → {Style[Text["m12"], FontSize → 12],
     Style[Text["m21"], FontSize → 12], None, None},
   FrameTicks → {{{0, 0.01, 0.02, 0.03}, None}, {{0, 0.01, 0.02, 0.03}, None}}];
```

```
fig4D = ListContourPlot[
   Flatten[Table[{S1, S2, branchinginv1weighted[0.01, 0.01, S1, S2, 0.1] +
       branchinginv2weighted[0.01, 0.01, S1, S2, 0.1]}, {S1, 0.01, 0.05, 0.001},
     {S2, 0.01, 0.05, 0.001}], 1], Contours → Table[i / 800, {i, 0, 12}],
   ColorFunction → ColorData[{"BlueGreenYellow", {0, 0.015}}],
   ColorFunctionScaling → False, PlotLegends → None, ImageSize → Small,
   Epilog → {Text[Style["D", 14, White], {0.013, 0.0475}]},
   FrameLabel → {Style[Text["s₁"], FontSize → 12],
     Style[Text["s₂"], FontSize → 12], None, None},
   FrameTicks → {{{0, 0.01, 0.02, 0.03, 0.04, 0.05}, None},
     {{0, 0.01, 0.02, 0.03, 0.04, 0.05}, None}}];
]
```

In[ ]:=
```
legend = BarLegend[{"BlueGreenYellow", {-0.0001, 0.01501}},
   Table[i / 800, {i, 0, 12}], LegendMarkerSize → 300,
   LegendLabel → "γ11 (A,B) \n Γ (C,D)", Ticks → {0, 0.005, 0.01, 0.015}];
Legended[Grid[{{fig4A,
     fig4B}, {fig4C,
     fig4D}}], legend]
```

Out[ ]=

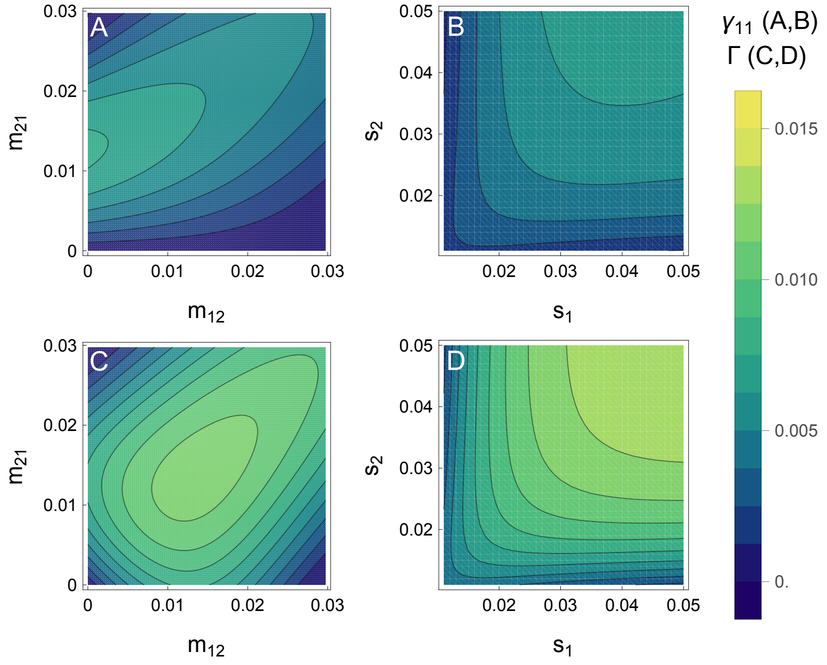

Supplementary

Approximation comparisons

Compare the performance of each approximation and the numerical solution --- the parameters used can be read from the code used to generate the figure (e.g. m -> 0.001)

In[ ]:=
```
DiscretePlot[
 {CIinvAdvNum[0.001, 0.01, R], m (2 m + r - 2 s)/r /. m → 0.001 /. s → 0.01 /. r → R,
  (m r) / (r + 2 s - 2 m) /. m → 0.001 /. r → R /. s → 0.01,
  (m r) / (r + 2 s) /. m → 0.001 /. r → R /. s → 0.01},
 {R, 0.02, 0.5, 0.001}, Joined → True, Filling → None,
 PlotLegends → {"numerical", "QLE", "Charlesworth & Barton", "weak m"},
 PlotRange → Full, ImageSize → Medium, AxesStyle → Thick, AxesLabel → Automatic]
```

Out[ ]=

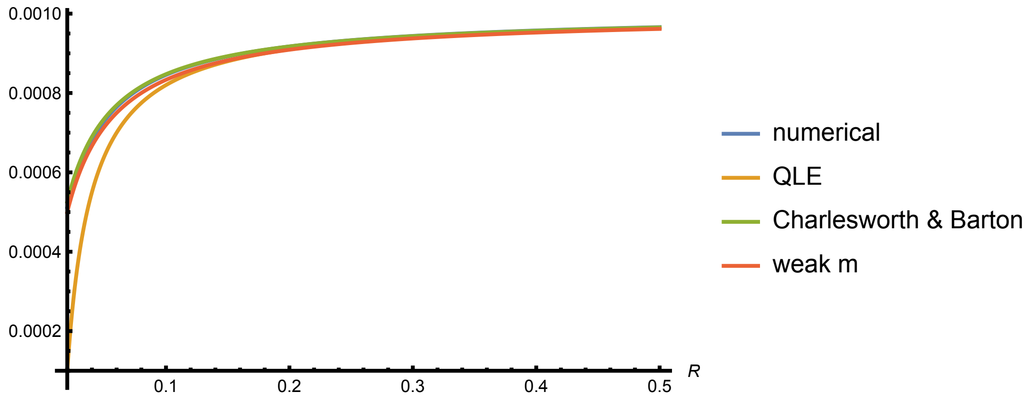

In[ ]:=
```
DiscretePlot[
 {CIinvAdvNum[0.03, 0.05, R], m (2 m + r - 2 s)/r /. m → 0.03 /. s → 0.05 /. r → R,
  (m r) / (r + 2 s - 2 m) /. m → 0.03 /. r → R /. s → 0.05,
  (m r) / (r + 2 s) /. m → 0.03 /. r → R /. s → 0.05},
 {R, 0.02, 0.5, 0.001}, Joined → True, Filling → None,
 PlotLegends → {"numerical", "QLE", "Charlesworth & Barton", "weak m"},
 PlotRange → Full, ImageSize → Medium, AxesStyle → Thick, AxesLabel → Automatic]
```

Out[ ]=

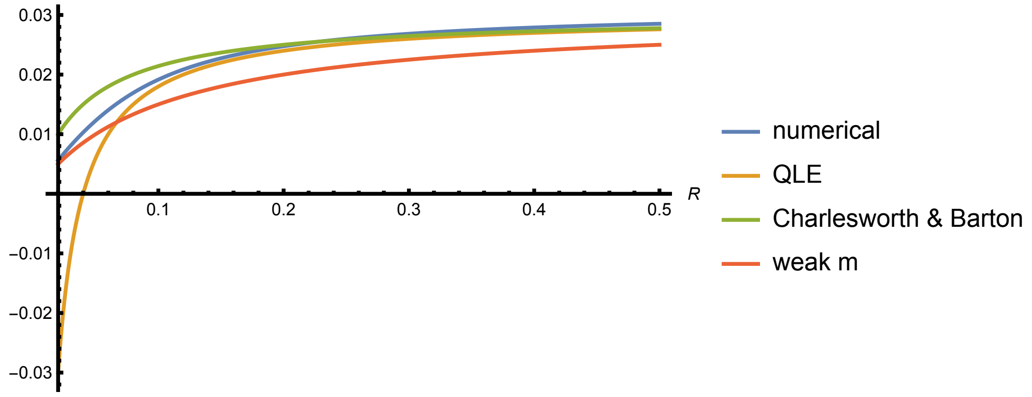

In[ ]:=
```
DiscretePlot[
 {CIinvAdvNum[0.001, S, 0.25], m (2 m + r - 2 s)/r /. m → 0.001 /. s → S /. r → 0.25,
  (m r) / (r + 2 s - 2 m) /. m → 0.001 /. r → 0.25 /. s → S,
  (m r) / (r + 2 s) /. m → 0.001 /. r → 0.25 /. s → S},
 {S, 0.001, 0.1, 0.005}, Joined → True, Filling → None,
 PlotLegends → {"numerical", "QLE", "Charlesworth & Barton", "weak m"},
 PlotRange → Full, ImageSize → Medium, AxesStyle → Thick, AxesLabel → Automatic]
```

Out[ ]=

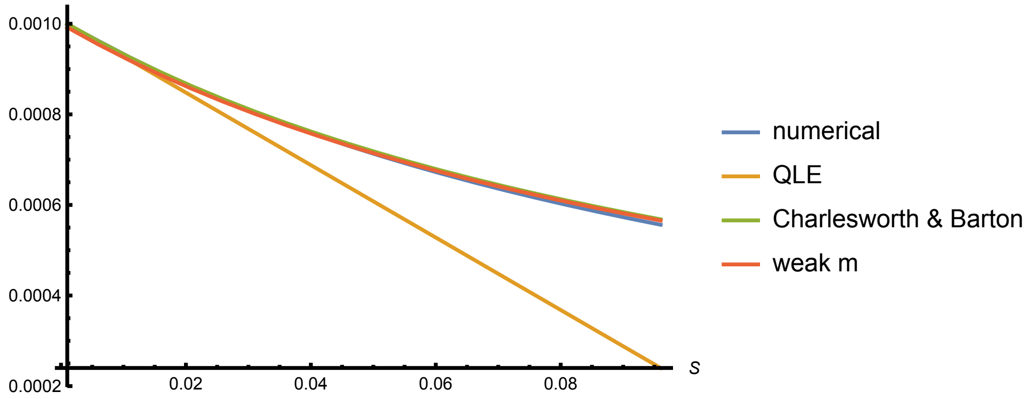

In[ ]:=
```
DiscretePlot[
 {CIinvAdvNum[0.01, S, 0.25], m (2 m + r - 2 s)/r /. m → 0.01 /. s → S /. r → 0.25,
  (m r) / (r + 2 s - 2 m) /. m → 0.01 /. r → 0.25 /. s → S,
  (m r) / (r + 2 s) /. m → 0.01 /. r → 0.25 /. s → S},
 {S, 0.01, 0.1, 0.001}, Joined → True, Filling → None,
 PlotLegends → {"numerical", "QLE", "Charlesworth & Barton", "weak m"},
 PlotRange → Full, ImageSize → Medium, AxesStyle → Thick, AxesLabel → Automatic]
```

Out[ ]=

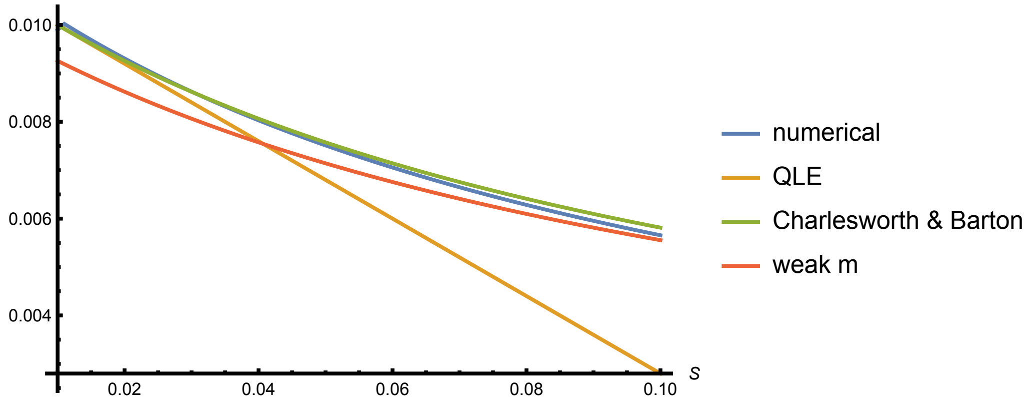

In[ ]:=
```
DiscretePlot[
 {CIinvAdvNum[M, 0.05, 0.25], (m (2 m + r - 2 s))/r /. m → M /. s → 0.05 /. r → 0.25,
  (m r) / (r + 2 s - 2 m) /. m → M /. r → 0.25 /. s → 0.05,
  (m r) / (r + 2 s) /. m → M /. r → 0.25 /. s → 0.05},
 {M, 0.0, 0.05, 0.001}, Joined → True, Filling → None,
 PlotLegends → {"numerical", "QLE", "Charlesworth & Barton", "weak m"},
 PlotRange → Full, ImageSize → Medium, AxesStyle → Thick, AxesLabel → Automatic]
```

Out[ ]=

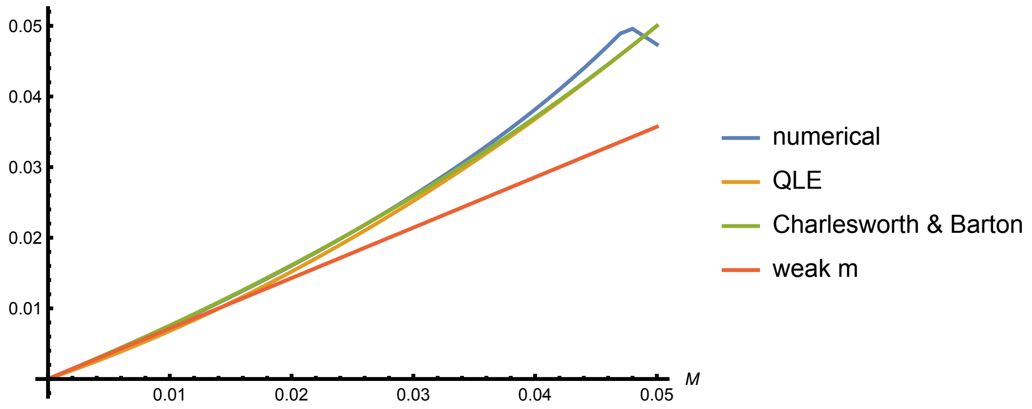

In[ ]:=
```
DiscretePlot[
 {CIinvAdvNum[M, 0.05, 0.1], (m (2 m + r - 2 s))/r /. m → M /. s → 0.05 /. r → 0.1,
  (m r) / (r + 2 s - 2 m) /. m → M /. r → 0.1 /. s → 0.05,
  (m r) / (r + 2 s) /. m → M /. r → 0.1 /. s → 0.05},
 {M, 0.0, 0.05, 0.001}, Joined → True, Filling → None,
 PlotLegends → {"numerical", "QLE", "Charlesworth & Barton", "weak m"},
 PlotRange → Full, ImageSize → Medium, AxesStyle → Thick, AxesLabel → Automatic]
```

Out[ ]=

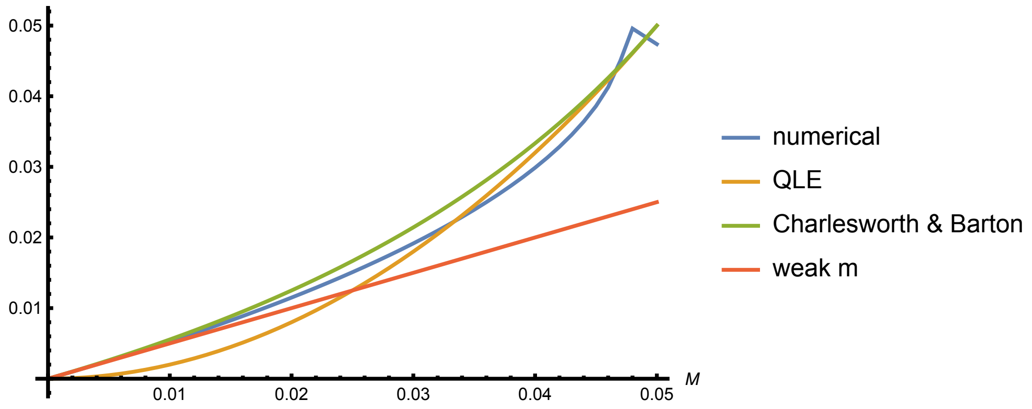

Approximate branching process solution

Try to find an approximate solution to the pair of branching process equations:

```
In[ ]:= a11 = FullSimplify[
    mat⟦1, 1⟧ /. wbar1 → p1Wm /. wbar2 → p2Wm /. wSubs /. alleleSubs /. rsubs /.
        freqFirstOrderTermSubs /.
      freqLinearTermSubs /. qleD /. symmetrySubs, s > 0];
a12 = FullSimplify[
    mat⟦1, 2⟧ /. wbar1 → p1Wm /. wbar2 → p2Wm /. wSubs /. alleleSubs /. rsubs /.
        freqFirstOrderTermSubs /.
      freqLinearTermSubs /. qleD /. symmetrySubs, s > 0];
a21 = FullSimplify[
    mat⟦2, 1⟧ /. wbar1 → p1Wm /. wbar2 → p2Wm /. wSubs /. alleleSubs /. rsubs /.
        freqFirstOrderTermSubs /.
      freqLinearTermSubs /. qleD /. symmetrySubs, s > 0];
a22 = FullSimplify[
    mat⟦2, 2⟧ /. wbar1 → p1Wm /. wbar2 → p2Wm /. wSubs /. alleleSubs /. rsubs /.
        freqFirstOrderTermSubs /.
      freqLinearTermSubs /. qleD /. symmetrySubs, s > 0];
```

In[ ]:=
```
A11 = Simplify[
   Normal[Series[a11 /. r → r ϵ /. s → s ϵ^2 /. m → m ϵ^2, {ϵ, 0, 4}]] /. ϵ → 1, s > 0];
A12 = Simplify[
   Normal[Series[a12 /. r → r ϵ /. s → s ϵ^2 /. m → m ϵ^2, {ϵ, 0, 4}]] /. ϵ → 1, s > 0];
A21 = Simplify[
   Normal[Series[a21 /. r → r ϵ /. s → s ϵ^2 /. m → m ϵ^2, {ϵ, 0, 4}]] /. ϵ → 1, s > 0];
A22 = Simplify[
   Normal[Series[a22 /. r → r ϵ /. s → s ϵ^2 /. m → m ϵ^2, {ϵ, 0, 4}]] /. ϵ → 1, s > 0];

Normal[Series[Exp[-(A11 u1 + A12 u2)] /. r → r ϵ /. s → s ϵ^2 /. m → m ϵ^2 /.
      u1 → u1 ϵ^2 /. u2 → u2 ϵ^3, {ϵ, 0, 5}]] /. ϵ → 1 // Simplify;
Normal[Series[Exp[-(A21 u1 + A22 u2)] /. r → r ϵ /. s → s ϵ^2 /. m → m ϵ^2 /.
      u1 → u1 ϵ^2 /. u2 → u2 ϵ^3, {ϵ, 0, 5}]] /. ϵ → 1 // Simplify;

Solve[{%%, %} == {1 - u1, 1 - u2}, {u1, u2}] // FullSimplify;

usubs = %[[2]]
```

Solve : There may be values of the parameters for which some or all solutions are not valid.

Out[ ]=

$$\left\{u1 \to \frac{4}{9}\left(\frac{36\,m^2}{r} + \frac{6\,m\,s + 9\,s^2 - 9\,m\sqrt{4\,m^2+s^2} - 9\,s\sqrt{4\,m^2+s^2}}{3\,m + 2\,s} + m\left(6 - \frac{9\left(8\,m^2+s^2\right)}{r\sqrt{4\,m^2+s^2}}\right)\right),\right.$$
$$u2 \to \Big(4\left(m - s + \sqrt{4\,m^2+s^2}\right)\Big(-24\,m^4 + r\,s^2\left(-s + \sqrt{4\,m^2+s^2}\right) +$$
$$4\,m^3\left(-r - 4\,s + 3\sqrt{4\,m^2+s^2}\right) - m\,s\left(2\,s^2 + r\left(s - 2\sqrt{4\,m^2+s^2}\right)\right) +$$
$$\left.m^2\left(2\,r\left(-2\,s + \sqrt{4\,m^2+s^2}\right) + s\left(-3\,s + 8\sqrt{4\,m^2+s^2}\right)\right)\Big)\Big) \Big/ \left(r\,(3\,m+2\,s)^2\sqrt{4\,m^2+s^2}\right)\right\}$$

In[ ]:=
```
Simplify[
  u1 * p1g11 / (p1g11 + p2g11)  + u2 * p2g11 / (p1g11 + p2g11) /. usubs /. wbar1 →
           p1Wm /. wbar2 → p2Wm /. wSubs /.
         alleleSubs /. rsubs /. freqFirstOrderTermSubs /.
      freqLinearTermSubs /. qleD /. symmetrySubs, s > 0];
approxInvasion = FullSimplify[
  Normal[Series[% /. r → r ϵ /. s → s ϵ^2 /. m → m ϵ^2, {ϵ, 0, 3}]] /. ϵ → 1]
```

Out[ ]=

$$\frac{1}{r\,(3\,m+2\,s)^2\left(4\,m^2+s^2\right)^{3/2}}\,4\Big(-96\,m^7 + 2\,r\,s^5\left(s - \sqrt{4\,m^2+s^2}\right) + 16\,m^6\left(-r - 9\,s + 3\sqrt{4\,m^2+s^2}\right) -$$
$$m\,s^4\left(4\,s^2 + r\left(-3\,s + \sqrt{4\,m^2+s^2}\right)\right) + 8\,m^5\left(9\,s\left(-2\,s + \sqrt{4\,m^2+s^2}\right) + r\left(-s + \sqrt{4\,m^2+s^2}\right)\right) +$$
$$m^2\,s^3\left(-7\,r\left(-2\,s + \sqrt{4\,m^2+s^2}\right) + s\left(-13\,s + 15\sqrt{4\,m^2+s^2}\right)\right) +$$
$$2\,m^4\,s\left(2\,r\left(5\,s + \sqrt{4\,m^2+s^2}\right) + s\left(-58\,s + 33\sqrt{4\,m^2+s^2}\right)\right) +$$
$$m^3\,s^2\left(-2\,r\left(-5\,s + \sqrt{4\,m^2+s^2}\right) + s\left(-48\,s + 49\sqrt{4\,m^2+s^2}\right)\right)\Big)$$

Other figures:

Comparison of the invasion probabilities obtained through the eigenvalue and the branching process

In[ ]:=
```
DiscretePlot[{((2 * (Max[Eigenvalues[
            mat /. wbar1 → p1Wm /. wbar2 → p2Wm /. wSubs /. symmetrySubs /.
              twoDemeHapFreqsNum[M, M, 0.05, 0.05, 0.1] /. m → M]] - 1)) /.
      s → 0.05 /. twoDemeHapFreqsNum[M, M, 0.05, 0.05, 0.1]),
   branchinginv1[M, M, 0.05, 0.05, 0.1] /. r → 0.1 /. s → 0.05 /.
    m → M},
  {M, 0.0, 0.05, 0.001}, Joined →
   True, Filling →
   None,
  PlotLegends → {"2*s_inv", "branching process"},
  PlotRange →
   {0, 0.016},
  AxesLabel → {"m", "invasion probability"}]
```

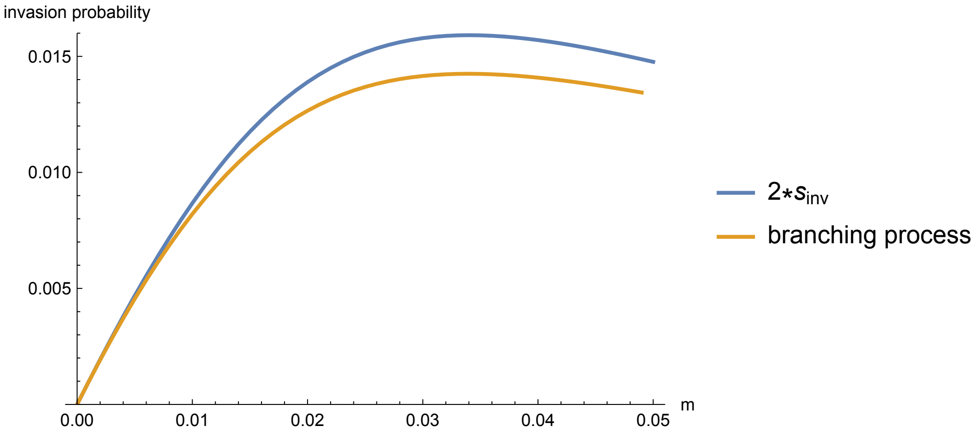

Plot the pattern of invasion probability when transitioning between symmetric and continent-island scenarios:

In[∘]:=
```
f2e = Labeled[DiscretePlot[{branchinginv1[M12, 0.02, 0.03, 0.03, 0.1],
     branchinginv1[M12, 0.02, 0.03, 0.03, 0.01]}, {M12, 0, 0.02, 0.001},
    Joined → True, Filling → None, PlotStyle → {{Thick, Black}, {Thick, Blue}},
    AxesStyle → Thick, ImageSize → Small, AxesLabel → {m12, p1},
    Ticks → {{0, 0.005, 0.01, 0.015, 0.02}, {0, 0.01, 0.02, 0.03, 0.04, 0.05}},
    PlotRange → {0.00, 0.04}],
   {Text["    CI                                        symm"]}, {{Bottom, Left}}];
f2f = Labeled[DiscretePlot[{branchinginv1[0.01, 0.01, 0.0100001, S2, 0.1],
     branchinginv1[0.01, 0.01, 0.0100001, S2, 0.01]}, {S2, 0.01, 0.05, 0.001},
    Joined → True, Filling → None, PlotStyle → {{Thick, Black}, {Thick, Blue}},
    AxesStyle → Thick, ImageSize → Small, AxesLabel → {s2, p1},
    Ticks → {{0.02, 0.03, 0.04, 0.05}, {0, 0.01, 0.02, 0.03, 0.04, 0.05}},
    PlotRange → {0.00, 0.02}],
   {Text["    symm                                        CI"]}, {{Bottom, Left}}];
Grid[{{%%, %}, {}}]
```

Out[∘]=

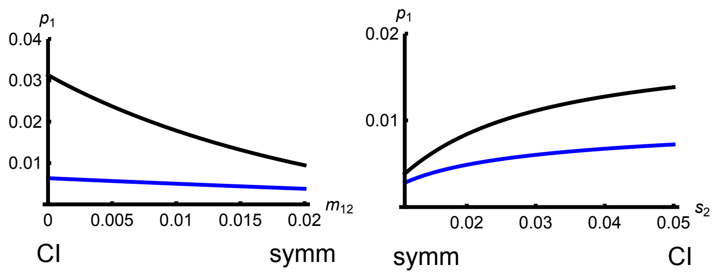

Supplement: iyae073_Supplementary_Data [file iyae073_supplementary_data.zip › File_S2_GENETICS-2024-306996.pdf]
