# Supplementary material for: Locally adaptive inversions in structured populations
Source: Genetics. 2024 May 6;227(3):iyae073. doi: 10.1093/genetics/iyae073 (PMC11979745; doi:10.1093/genetics/iyae073)
Supplement: iyae073_Supplementary_Data [file iyae073_supplementary_data.zip › Supplementary_Information_GENETICS-2024-306996.pdf]

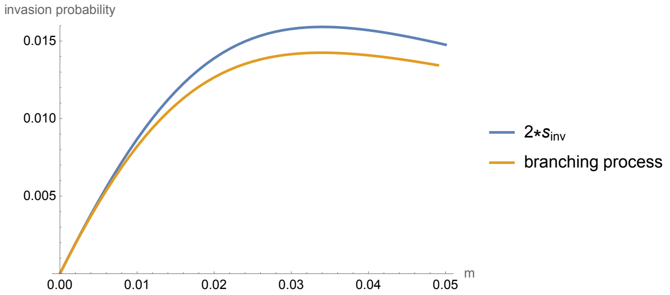

Figure S1 Probability of inversion invasion calculated from the asymptotic rate of increase s_{inv} and from the branching process. Parameter values are $s = 0.05, r = 0.1$. Data with $s < m$ are excluded as the adaptive alleles may not be at a stable equilibrium.

Supporting Information

Calculating invasion probabilities with a branching process

First, define the random variable $\mathbf{X}_l^k$ to be the number of offspring that, post-migration, are in deme l from a parent in deme k. Under Wright-Fisher conditions $\mathbf{X}_l^k$ has a Poisson distribution with mean c_{kl}, where c_{kl} is the k, l-th entry from the mean matrix $C_{A_1B_1}$ (Equation 3). Then, let $\mathbf{v} = (v_1, v_2)$, where v_i is the probability an inversion that starts in deme i is ultimately lost. In this case, $\mathbf{v}$ is the unique solution to the pair of equations

$$f^{(k)}(\mathbf{v}) = \mathbb{E}\left[v_1^{\mathbf{X}_1^k} v_2^{\mathbf{X}_2^k}\right] = \mathbf{v}, \tag{S1}$$

where $k \in \{1, 2\}, \mathbf{v} = (v_1, v_2) \in [0, 1]^2$ (Harris 2002). Since all the $\mathbf{X}_l^k$ are independent,

$$\mathbb{E}\left[v_1^{\mathbf{X}_1^k} v_2^{\mathbf{X}_2^k}\right] = \mathbb{E}\left[v_1^{\mathbf{X}_1^k}\right] \mathbb{E}\left[v_2^{\mathbf{X}_2^k}\right], \tag{S2}$$

in which each term in the product is the probability generating function of $\mathbf{X}_l^k$. It then follows that the two extinction probabilities are the solution to

$$\begin{aligned} v_1 &= e^{-(c_{11}(1-v_1)+c_{12}(1-v_2))}, \\ v_2 &= e^{-(c_{21}(1-v_1)+c_{22}(1-v_2))}. \end{aligned} \tag{S3}$$

The invasion probabilities $\mathbf{u} = (u_1, u_2)$ are thus given by $\mathbf{1} - \mathbf{v}$ (the probability of invasion is the probability of non-loss), and can be found numerically.

Literature cited

Harris TE. 2002. *The Theory of Branching Processes*. Dover Publications.
